# ANFIS-Net for automatic detection of COVID-19

**DOI:** 10.1038/s41598-021-96601-3

**Published:** 2021-08-27

**Authors:** Afnan Al-ali, Omar Elharrouss, Uvais Qidwai, Somaya Al-Maaddeed

**Affiliations:** Department of Computer Science and Engineering, Qatar University, Doha, Qatar

**Keywords:** Computer science, Infectious diseases

## Abstract

Among the most leading causes of mortality across the globe are infectious diseases which have cost tremendous lives with the latest being coronavirus (COVID-19) that has become the most recent challenging issue. The extreme nature of this infectious virus and its ability to spread without control has made it mandatory to find an efficient auto-diagnosis system to assist the people who work in touch with the patients. As fuzzy logic is considered a powerful technique for modeling vagueness in medical practice, an Adaptive Neuro-Fuzzy Inference System (ANFIS) was proposed in this paper as a key rule for automatic COVID-19 detection from chest X-ray images based on the characteristics derived by texture analysis using gray level co-occurrence matrix (GLCM) technique. Unlike the proposed method, especially deep learning-based approaches, the proposed ANFIS-based method can work on small datasets. The results were promising performance accuracy, and compared with the other state-of-the-art techniques, the proposed method gives the same performance as the deep learning with complex architectures using many backbone.

## Introduction

An epidemic is a disease that affects many people within a population or country, and it turns to a pandemic when it spreads widely around the world. This is the status of coronavirus that the World Health Organization (WHO) announced as a pandemic in 2019^1,2^. The high spreading nature of the virus makes the analysis time of the X-ray images or any other type of material used for diagnosis, a critical factor. This pushed the researchers to try their best to use the AI technology to propose an automated system to help the radiologist to speed up the process of diagnosis. Most popular techniques were used to achieve this purpose based on deep learning^3,4^. For their analysis, researchers have used different types of datasets like MRI, CT scan, and X-ray images^5^. X-ray images are considered the widely available type for image diagnosis, cheaper and faster than CT scan^6^. These medical images provide vague, uncertain, conflicting, distorted information which makes this type of data characterized with a confused structure^7^. Fuzzy Concept plays an important role in dealing with these kinds of information for many reasons: the fuzzy system can provide a realistic representation for the imprecise information in images like the imprecision of an object’s membership in a particular category, as well as, expressing this information at various stages (either local, global or regional) and different forms (either symbolic or numeric) with fuzzy sets. Finally, fuzzy framework is able to represent the heterogeneous information or even the features that are extracted directly from the images or provided from an outside expert^6,8^.

In this research paper, an ANFIS-Net based binary classification process has been implemented for automatically detecting COVID-19 infection from CXR images. In our proposal, we extracted six types of features using a statistical method from MATLAB named Gray level co-occurrence matrix (GLCM), where these features are used as an inputs to the Initial fuzzy Inference model, which is used later to run the adaptive neuro fuzzy inference system model for the classification process.

The organization of the rest of the paper is as the following: in section “Literature review”, a summary of the recent research works that have been done using ANFIS classifier in other medical fields and the recent research works that have been done on COVID-19 detection using other techniques is done. In section “Proposed method”, our proposed methodology has been presented. In section “Experimental results”, we described the experimental metrics and in section “Evaluation and discussion” all the results with discussion are explained in detail. Finally, in section “Conclusion” we conclude the paper.

## Literature review

Adaptive Neuro Fuzzy Inference System (ANFIS) classifier has many applications in medical field like detecting Delirium, dialysis, breast cancer, Brain tumor, autism, Alzheimer, blood diseases, kidney failure, chest diseases with the ability to deal successfully with both statistical datasets or Images (CT scan, chest X-ray). As there is no previous research on COVID-19 detection using this classifier, it seems fair enough to show its effectiveness by producing some of ANFIS classifier’s work in biomedical field.

For brain tumor detection^9–17^ ANFIS achieved great results with an accuracy range of 96–99%, and for bone cancer detection^18^ the accuracy was 93%. Breast cancer detection^19–22^, the detection was never better and the ANFIS classifier achieved an accuracy of the range 91–99%. Finally, in chest diseases detection^23,24^,the performance accuracy was around 98%.

Currently, there is no existing classification on COVID-19 done by researchers using fuzzy system, rather it was used to predict an infected, individuals time series and the rate of deaths. As in^25^ an improved model of ANFIS was proposed using both an enhanced flower pollination algorithm (FPA) and salp swarm algorithm (SSA) to predict the confirmed cases of COVID-19 within ten days. FPASSA-ANFIS performed better then the state-of-the-art prediction models in terms of all the evaluation metrics used for their tests.

A study of risk assessment of COVID-19^26^ was proposed based on Fuzzy logic prediction system. It was successfully demonstrated to take into account the changes in risk over time.

In^27^ an adaptive Neuro Fuzzy Inference System (ANFIS) classifier and multi-layered perceptron-imperialist competitive algorithm (MLP-ICA) was proposed to estimate the time series and the death rate of infected individuals.

Recent COVID-19 researches are either providing aid techniques for protection or for early diagnosis.Many significant studies are adopted for the first group like in telehealth services^28^, wearable technology^29^, breathing aid devices^30^ and detection tool of unwearable mask people^31^.The other group also adopted some successful experiments for diagnosis and automated detection mostly depends on deep learning techniques. In^32^a model of deep learning was proposed for automatic detection of coronavirus from X-ray datasets and classify them for both binary and multi class using Dark Net model which consists of 17 CNN and YOLO object detection system. A drawback of this technique is the limitations of COVID-19 CXR images.

Another deep CNN model in^33^ was proposed for detecting COVID-19 from X-ray images by building a huge dataset of 13,975 CXR images named COVIDx dataset to evaluate their model which was among the first open access resource for detecting COVID-19 from X-ray images by designing an initial prototype using lightweight residual projection-expansion projection-extension (PEPX) design pattern for analyzing the crucial features of the virus. Here the dataset is unbalanced with only 358 CXR images of COVID-19 compared with nearly 13,000 CXR images of the other two classes (pneumonia and Normal).

One more new framework of deep learning namely COVIDX-Net for diagnosing this virus from X-ray images was presented in^34^to assist radiologists. This system consists of seven different models of deep convolutional neural network they are: VGG19, DenseNet121, InceptionV3, ResNetV2, Inception-ResNet-V2, Xception, and MobileNetV2, where the accuracy was found to be around 60% to 90% and this still needs to be improved.

**Figure 1:**
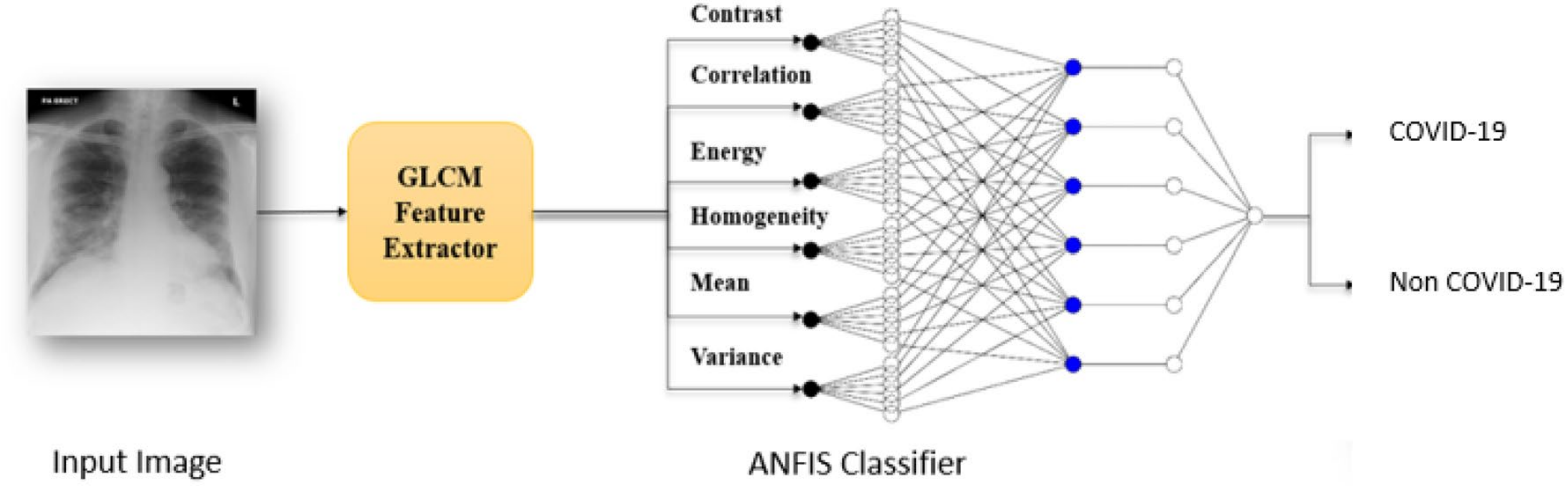
The workflow of the proposed method.

In^35^ Deep transfer learning was proposed on the basis of the use of three forms of convolutionary neural networks (ResNet50, InceptionV3 and Inception- ResNetV2) using CXR images for automatic prediction of COVID-19 patients where ResNet50 showed the best classification results among the rest with accuracy of 98%.Despite the good performance, the dataset was very small with only 50 CXR images for both classes (COVID-19 vs Non-COVID). Another example of transfer learning in^36^ which was a combination between CNN and RNN achieved high accuracy reached to 99.9 based on VGG19-RNN architecture for three classes (COVID, Pneumonia, non COVID), nevertheless, the dataset need to be updated as it is very small. For more efficient computing^37^, proposed a light CNN model with shallow structure to automatically detect COVID-19. The accuracy was promising compared with the state of the art at the time the research was done. However, the data set was small as well.

A combined model of deep learning and simple CNN was demonstrated^38^to be applied on mixed data set of CXR images and CT scan with an accuracy which reached to 94.4%, that can be improved more if the dataset was larger. A comparison among 9 fined tuned pre-trained models was demonstrated for automatic detection in pneumonia datasets^39^. This comparison was applied on mixed data set as well for CT scan and CXR images and the accuracy was around 80%-90%.

There are some other techniques other than deep learning which were used for the automatic detection of COVID-19 like in^40^ where an ensemble classifier was used as an automatic screening system for diagnosing COVID-19 from the chest X-ray where the accuracy was around 98% but was tested on small size dataset. Another ensemble classifier was tested in^41^ combining four binary machine learning classifiers (random forest, support vector machine, decision tree, and AdaBoost) with accuracy reached to 98.91%. Also, a kernel and linear kernel SVM classification model was developed in^42^ to carry out 2 classifications (COVID and normal) and 3 classifications (COVID, pneumonia and normal) after a feature extraction procedure using several forms of convolutionary neural network. The results were promising which reached to 100% with 2 classes and 97.3% with 3 classes but still the slow processing time with neural network is the weakness here.

Another research that used CNN as feature extractor is in^43^ combining this network with Long-Short term memory (LSTM) for automatic diagnosis of COVID-19 from chest X-ray data set with accuracy 99.4%.

With this it is clear that there’s a need to test new model trained on a large dataset with better accuracy to detect COVID-19 which is explored here.

## Proposed method

Several classification methods have been proposed for automatic detection of COVID-19 since it started, and almost all of them were built upon deep learning models. We chose to use the Fuzzy logic idea in this research for two reasons:

The first is that most concepts in medical field are fuzzy which make them difficult to measure or formalize and the fuzzy system seems a sufficient solution to deal with this type of dataset. The second reason is that, first part of the literature review showed how the Adaptive Neuro Fuzzy Inference system (ANFIS) classifier is effective in classification process with very high accuracy reaching to 99% and it is the time to use this classifier for COVID detection as this is the recent situation. The size of dataset used in related experiments of ANFIS in other medical fields were not larger than 500 images which is not the case of this experiment which has a very large dataset.

In this research study, we use the adaptive Neuro Fuzzy Inference framework based on subtractive clustering technique to detect COVID-19 from a large data set of X-ray images. We used a Gray level Co-occurrence mathematical technique to extract the spatial features between image pixels, where four features are extracted: correlation, contrast, energy and homogeneity. We add two more features, they are mean and variance calculated from the image histogram. These six features were calculated for each image representing a raw image dataset for usefulness of accurate classification decision. After feature extraction, the dataset is prepared to be used as an input to the ANFIS -Net to be classified. The flow chart below in Fig. 1 describes the steps to accomplish this objective. And the latter subsections describes each move in more details.

### Feature extraction step

For pre-processing, each image in the data set is converted to gray scale and resized to $512 \times 512$ then they were normalized to decrease the computational complexity.

To extract the required features, a gray-level co-occurrence matrix (GLCM) technique is performed. It is a mathematical method that considers the spatial pixel relationship in the image. This function computes how often a specific value of a pair of pixels occurring in an image (this characterizes the texture of the image)^18,19^. This computing occurs within 45 degree interval in four specific directions: 0, 45, 90 and 135 and scalar distance (number of neighbour pixels). The first step of the GLCM algorithm is to calculate the GLCM matrix from the test image by initializing a two pixel GLCM matrix in any of the previously mentioned directions, and then generating a symmetric matrix by summing the latter with its transpose. Finally, divide each element of this matrix by the number of pixel pairs to get the normalized version of GLCM matrix. From this final version, the correlation, contrast, energy and homogeneity features are calculated^44^. Two more features, that are mean and variance were computed from the histogram distribution of each image representing another statistical information included in the image^45^. Algorithm 1 summarizes the procedure of feature extraction from GLCM matrix and from the image. The first four features are extracted from GLCM, they are: The Contrast (represents a measure of the intensity contrast between each pixel and its neighbor over full image), the Correlation (represents a measure of pixel correlation with its neighbor over the whole image),the Energy (it is the total amount of the GLCM square parameters) and Homogeneity (the distributed similarity of the elements in the GLCM to its diagonal). Mean and variance features are extracted from the histogram of the image, where mean is the image’s intensity average value and variance represents the way the pixels are spread^18,45,46^.
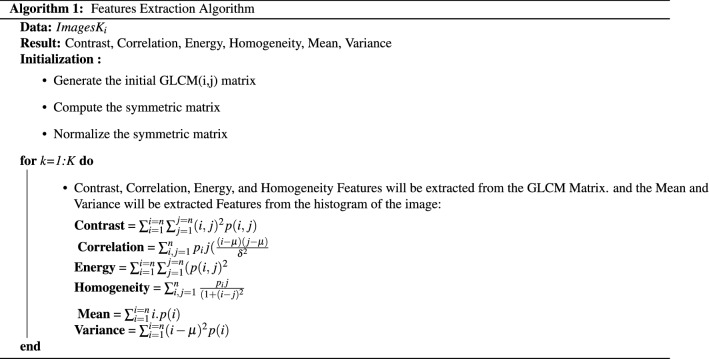


Here i, j in Algorithm 1 are the rows and columns respectively of the GLCM matrix and p (i, j) demonstrates the relative position of the pixels in the matrix. $\delta$ and $\mu$ depict the variance and the mean of the image itself. respectively^18,45^.

### Classification step

After the feature extraction step, a subtractive clustering technique based on neuro fuzzy logic classifier is adopted. The next sub sections will explain the structure of this classifier and the detail of its technique.

#### ANFIS classifier structure

**Figure 2:**
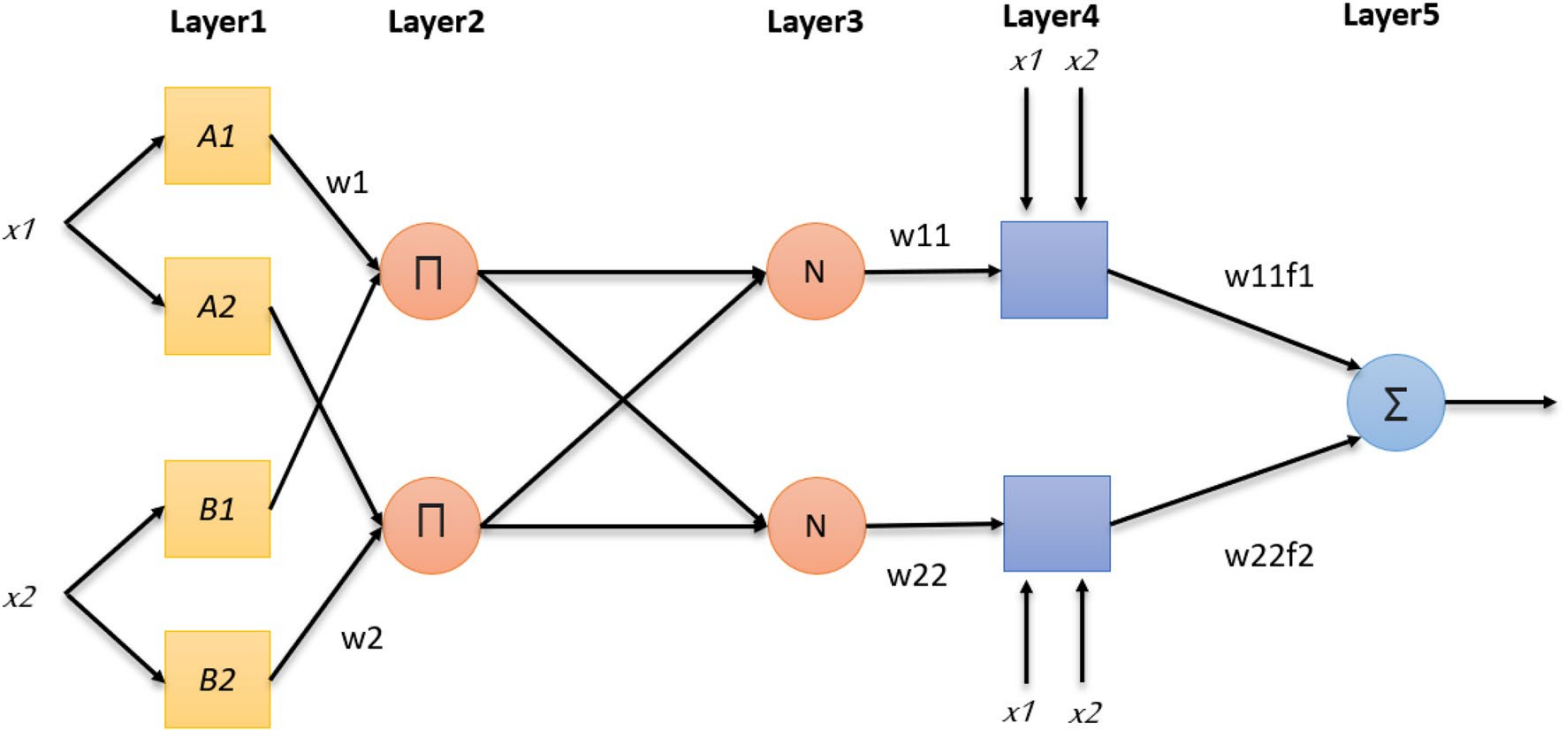
The structure of 2 inputs parameters of ANFIS.

The extracted features are applied to the adaptive neuro fuzzy inference system. ANFIS classifier is a mixture of neural and fuzzy inference system networks^18^ and its architecture is constructed by five layers as shown in Fig. 2^45,47^.

The input values enters the first layer (The Fuzzy layer) which determines the membership functions belonging to each one, that’s why it is called fuzzification layer. In this step each input will have its own group of membership function or the set that it is transformed to, for example

1 $$\begin{aligned} f1=\{(p_1.x+q_1.y+r_1), x==A_1,y==B_1\} \end{aligned}$$

here x and y are the initial membership functions, A1 and B1 represent the values of these memberships. f1 is the output and the consequent parameters are p1,q1 and r1.These membership functions are computed by using the premise parameters a, b, c

The rules layer is the second layer which generates the firing strengths for the rules.The outcome of every node is represented by the product of all incoming signals^47^.

The third layer is the normalization layer, its role is to normalize these firing strengths by computing their sum and dividing each value on it

2 $$\begin{aligned} W_{11}= & {} \frac{W_1}{{W_{1}}+{W_{2}}} \end{aligned}$$

3 $$\begin{aligned} W_{22}= & {} \frac{W_2}{{W_{1}}+{W_{2}}} \end{aligned}$$

The fourth layer is the defuzzy layer that takes the normalized values as input as well as the consequence parameters p, q, r. In this fuzzy system, the output is the weighted sum of their inputs added by a constant. The output of this layer is the defuzzification parameters.

4 $$\begin{aligned} O_i=w_i f_1=w_i (p_i x+q_i y+r_i), \quad i=1,2 \end{aligned}$$

The fifth layer (The output layer) computes the average of each rule output giving the final output of the system.

5 $$\begin{aligned} \sum _{i=1}^{i=n} W_if_i= \frac{\sum _{i=1}^{i=n} W_if_i}{\sum _{i=1}^{i=n} W_i} \end{aligned}$$

**Figure 3:**
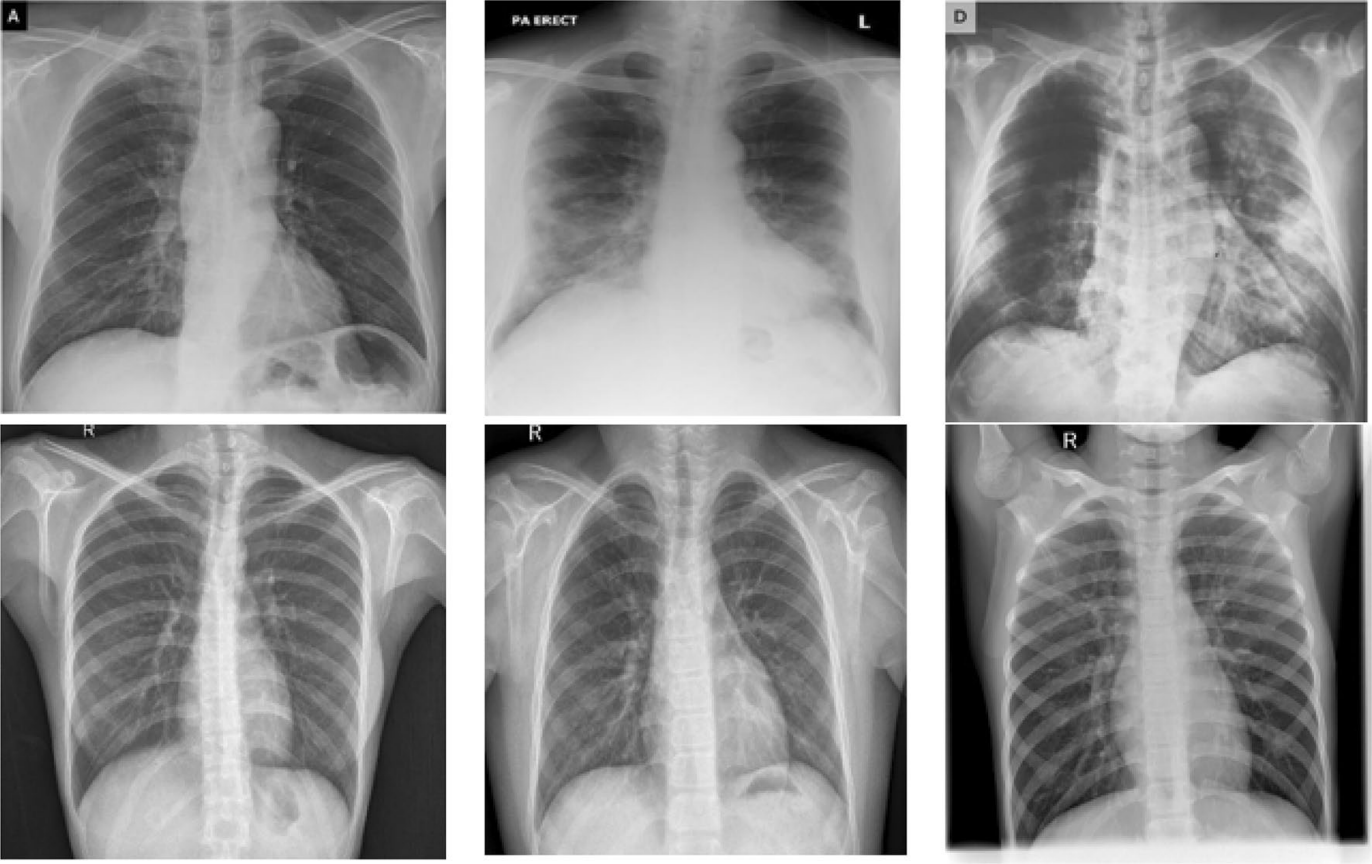
Sample of the dataset. The first row shows sample of COVID-19 cases and the second row shows the normal cases.

The default optimization algorithm for ANFIS parameters during the training part of this classification process is the hybrid learning algorithm. It is a mixture of two passes of back propagation and least squares methods: forward and backward. Each pass is used to update a type of parameter^24^. In the forward path, the premise parameters are modified using the gradient decent and during the backward pass the consequent variables are updated and the performance depends on them^48^. The selected features are provided as inputs to the first layer to be trained by ANFIS classifier during the training mode. The output of this part is a trained element ‘f’. During the testing part, this process is repeated on the features of the test dataset and produces a binary output corresponding to the classes from the fifth layer^45,47^.

#### Subtractive clustering technique

There are two methods to implement the fuzzy parameters of ANFIS in MATLAB, the grid partitioning and subtractive clustering. The grid partitioning is the default method which used to generate rules based on the given membership function and divide the data set into arbitrary partition. For each membership function, a rule will be generated, so that if the total numbers of variables increase, the generated rules will be increased exponentially causing a problem of dimensionality and this technique is sufficient for small data set^24,48,49^.

On the other hand, subtractive clustering is another technique where each data point is labeled as a cluster center and the number of generated rules will be independent on the number of variables and will be linear to the data points^49,50^. For each data point, the probability of the frequency of the neighbors data points will be determined, allowing the high potential to be chosen as the first cluster core. The influence range (cluster radius) is then used to delete all data points in the range of the first cluster core (the usually used values are between 0.2 and 0.5). The smaller ranges of this factor will produce more clusters.This process is repeated until all the cluster centers are chosen within the range of influence. There is also squash factor which is used to scale the range of influence and in order to generate more and small clusters,a small value of squash factor should be chosen because this will reduce the ability to consider the outlying points as part of the cluster (usually 1.25). Accept ratio is also used to consider all the data points above the first cluster center and reject ratio is defined as the value which is below the first cluster, is rejected as a cluster point, both these two later values are between 0 and 1 and the accept ratio should be higher than the reject value^50,51^.

## Experimental results

In this research study, we tested the impact of subtractive clustering parameters on classification process by applying 12 tests starting from the default values until we reached the best results in test 12 with 5 folds cross validation to avoid over fitting, as we will explain later in detail, and then compared our work with some of the state of the art techniques.

### Data set description

The dataset which used in our experiment is the updated version of the dataset mentioned in^43^, but for our experiment of binary classification, we only selected two classes that consist of 2000 Images, 1000 of COVID-19 and 1000 of non COVID-19 and for this analysis, it is divided into 70% training (1400 images) and 30% for testing (600 images), Fig. 3 shows sample of the dataset.

### Experimental setup

The proposed model is implemented using a computer with the following specifications: OS- Microsoft Windows 10 Home Processor- Intel (R) Core (TM) i7-7500U CPU @ 2.70 GHz, (RAM)- 12.0 GB The training and test are done using MATLAB R2020a by using MATLAB Fuzzy Logic Toolbox (FLT) from MathWorks as an implementation tool.

### Evaluation metrics

The accuracy of the model output is determined as the following equation to verify the performance of our model.

6 $$\begin{aligned} Accuracy=\frac{TP+TN }{TP+TN+FP+FN}, \end{aligned}$$

where TP or (True Positive): describes the correctly labeled as positive cases of COVID-19. TN or (True Negative): describes the correctly labeled as negative cases of COVID-19 (Normal). FP or (False positive): describes the Normal cases that labeled as COVID-19. FN or (False Negative): describes the COVID-19 cases that labeled as Normal.

Two more evaluation metrics are used, they are:

- sensitivity: it is the capability of our model to efficiently recognize the positive cases of COVID-19
- specificity: it is the capability of our model to efficiently recognize the Normal cases

7 $$\begin{aligned} Sensitivity= & {} \frac{TP}{TP+FN} \end{aligned}$$

8 $$\begin{aligned} Specificity= & {} \frac{TN}{TN+FP} \end{aligned}$$

**Figure 4:**
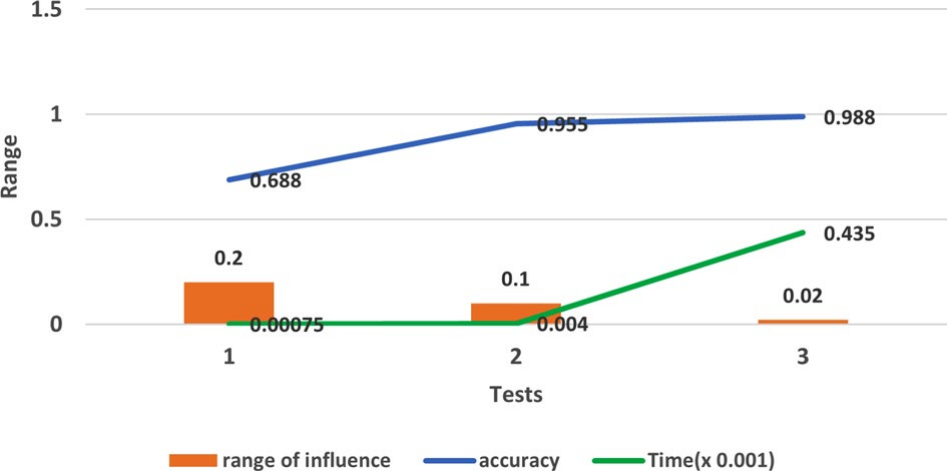
The impact of range of influence on accuracy in tests 1, 2 and 3.

**Figure 5:**
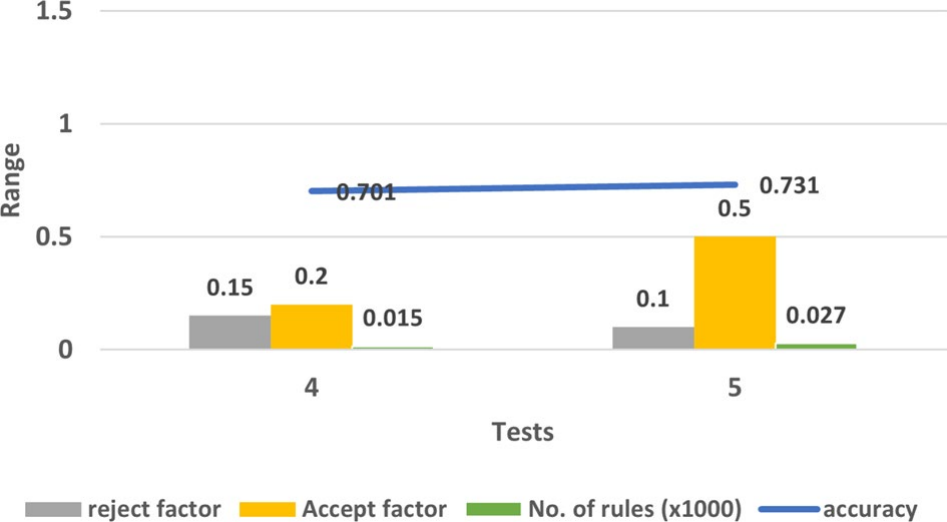
The impact of accept factor and reject factor on accuracy in test 4 and 5.

## Evaluation and discussion

To analyze the performance of ANFIS for the classification process, 12 tests have been done to check the effect of the subclustering parameters to get a high accuracy without over-fitting the data and in short period of time. Table 1 shows the selected values for each test with its corresponding performance results.

**Table 1:** The performance metrics of ANFIS classifier.

Tests	Subtractive clustering parameters				No. of rules	Evaluation metrics			Time (in min)
	Range of influence	Squash factor	Accept factor	Reject factor		Accuracy	Sensitivity	Specificity	
Test 1	0.2	1.25	0.5	0.15	17	0.688	0.662	0.728	50 sec
Test 2	0.1	1.25	0.5	0.15	162	0.955	0.953	0.956	4
Test 3	0.02	1.25	0.5	0.15	1190	**0.988**	**0.99**	**0.996**	**435**
Test 4	0.2	1.25	0.2	0.15	15	0.701	0.715	0.687	1.5
Test 5	0.2	1.25	0.5	0.1	166	0.731	0.721	0.742	4
Test 6	0.2	1.2	0.5	0.15	16	0.688	0.681	0.687	< 1
Test 7	0.2	1.1	0.5	0.15	23	0.738	0.741	0.743	< 1
Test 8	0.2	0.9	0.5	0.15	32	0.768	0.765	0.772	< 1
Test 9	0.2	0.25	0.5	0.15	163	0.906	0.914	0.902	5
Test 10	0.2	0.25	0.2	0.1	422	0.950	0.953	0.946	60
Test 11	0.1	0.25	0.2	0.1	496	0.978	0.967	0.986	71
Test 12	0.1	0.1	0.2	0.1	529	**0.986**	**0.989**	**0.984**	**30**

Bold values indicates the highest evaluation matrices we got from both test 3 and test 12.

In test 1, all the parameters’ values used are default and it shows low accuracy about 0.688, while the generated rules were only 17; the only advantage is the short processing time. In test 2 and test 3, we reduced the radius of the clusters by reducing the range of influence to generate more clusters and the performance improved rapidly to be 0.955 in test 2. which inspired us to reduce more, so we got a significant improvement in performance to reach almost 0.998 in test 3, but the drawback in test3 is that it lasts for more than 6 hours (for both training and testing) which is very long time and unpractical. Figure 4 shows the impact of reducing the range of influence on accuracy starting from test 1, 2 and 3.

Test 4 and 5 were about checking the effect of decreasing the values of accept factor and reject factor and it seems from the testing results that neither reducing the accept factor nor the reject factor produce a noticeable effect on performance. and the accuracy is around 0.70 to 0.73 which is better than the accuracy when using the default values in test 1 but it can be improved more. Figure 5 shows the impact of changing these two factors on accuracy.

**Figure 6:**
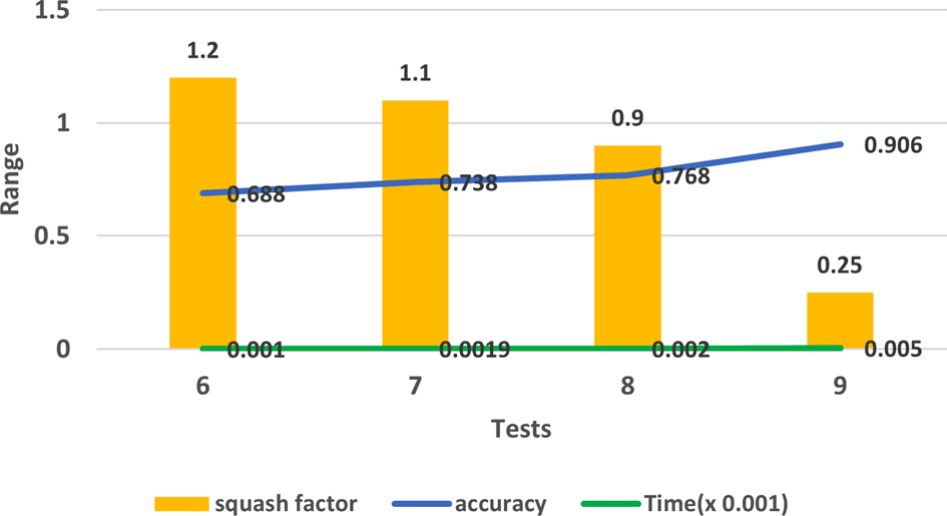
The impact of squash factor on accuracy in tests 6, 7, 8 and 9.

**Figure 7:**
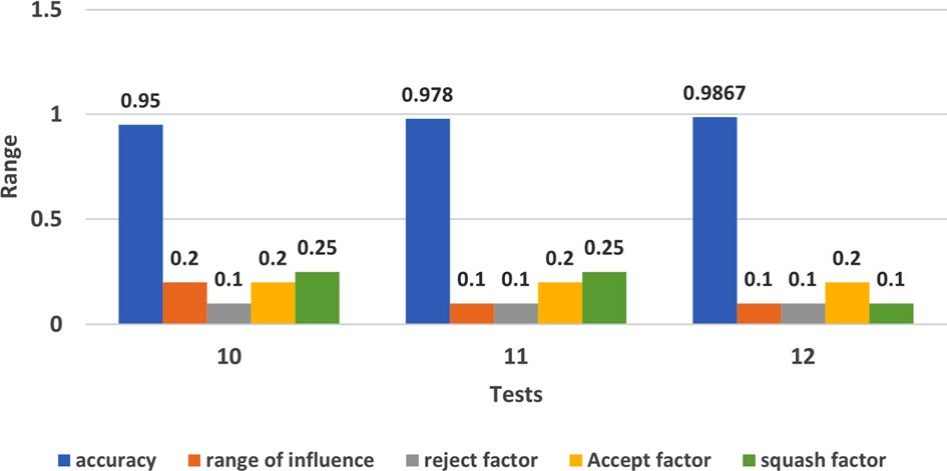
The best parameters values from previous test in tests 10, 11 and 12.

From test 6 till test 9, we checked the effect of squash factor while keeping the default values for the rest of the parameters. The model showed a progress in its performance and the processing time was also short, which is the goal of this experiment. Figure 6 displays this promising relation between Squash factor and model accuracy in these 4 tests. In test 10, we selected the best values of all the parameters from the previous tests and gathered them in one more experiment (0.2 for range of influence, 0.25 for squash factor, 0.2 for accept factor and 0.1 for reject factor), the accuracy was good and the processing time was acceptable, so in test 11, we added smaller value for the range of influence to be 0.1 as this factor has big effect on improving accuracy as we mentioned before, and the accuracy was promising with 0.978 as shown in Fig. 7.

As a final experiment, and in order to improve the accuracy more, we did one last test with the previous selected values (in test 11) but changed only the value of Squash factor as it is considered as the second effected factor on enhancing the findings of the model in case of large data set,to be 0.1. The accuracy we got from this test was the best compared with all the other tests to be 0.9867 in 30 min.

**Figure 8:**
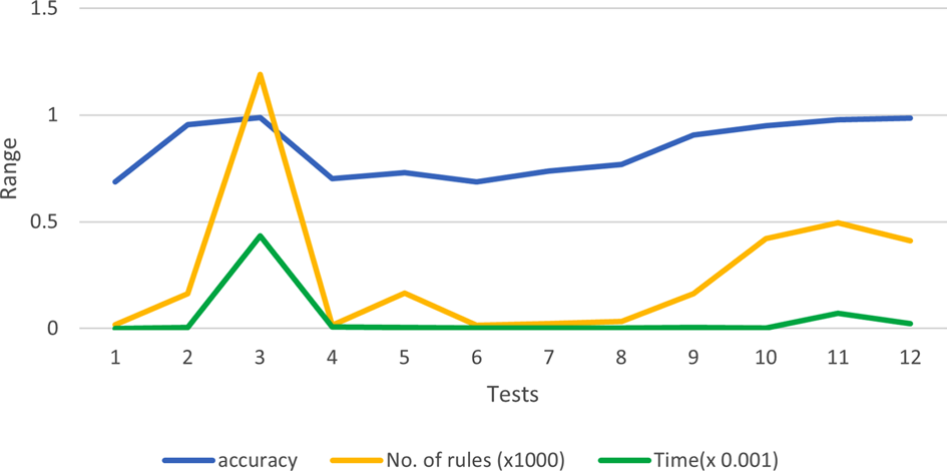
The relation between the generated rules and the processing time.

**Figure 9:**
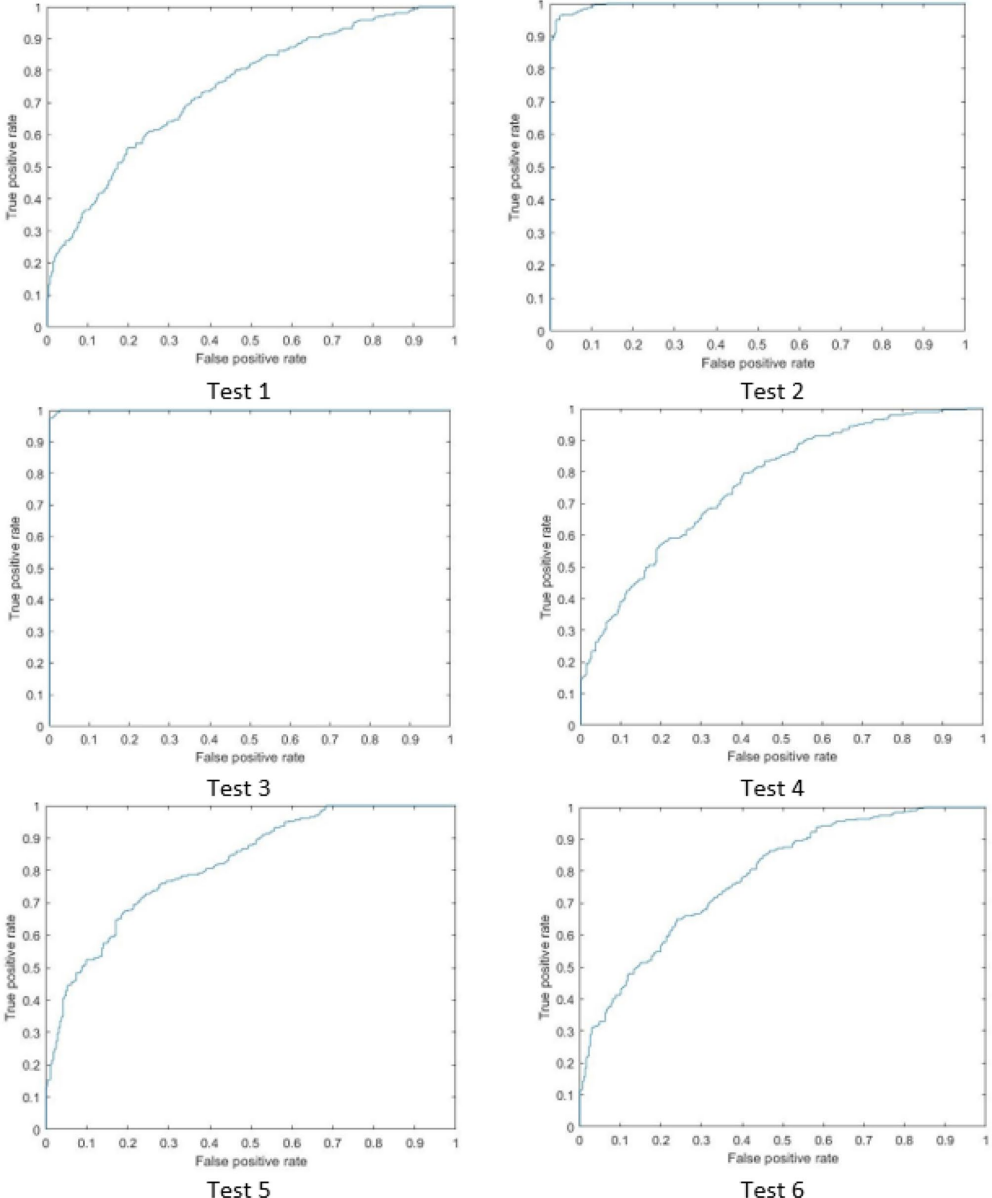
The ROC curve of the first six tests in our proposed method.

**Figure 10:**
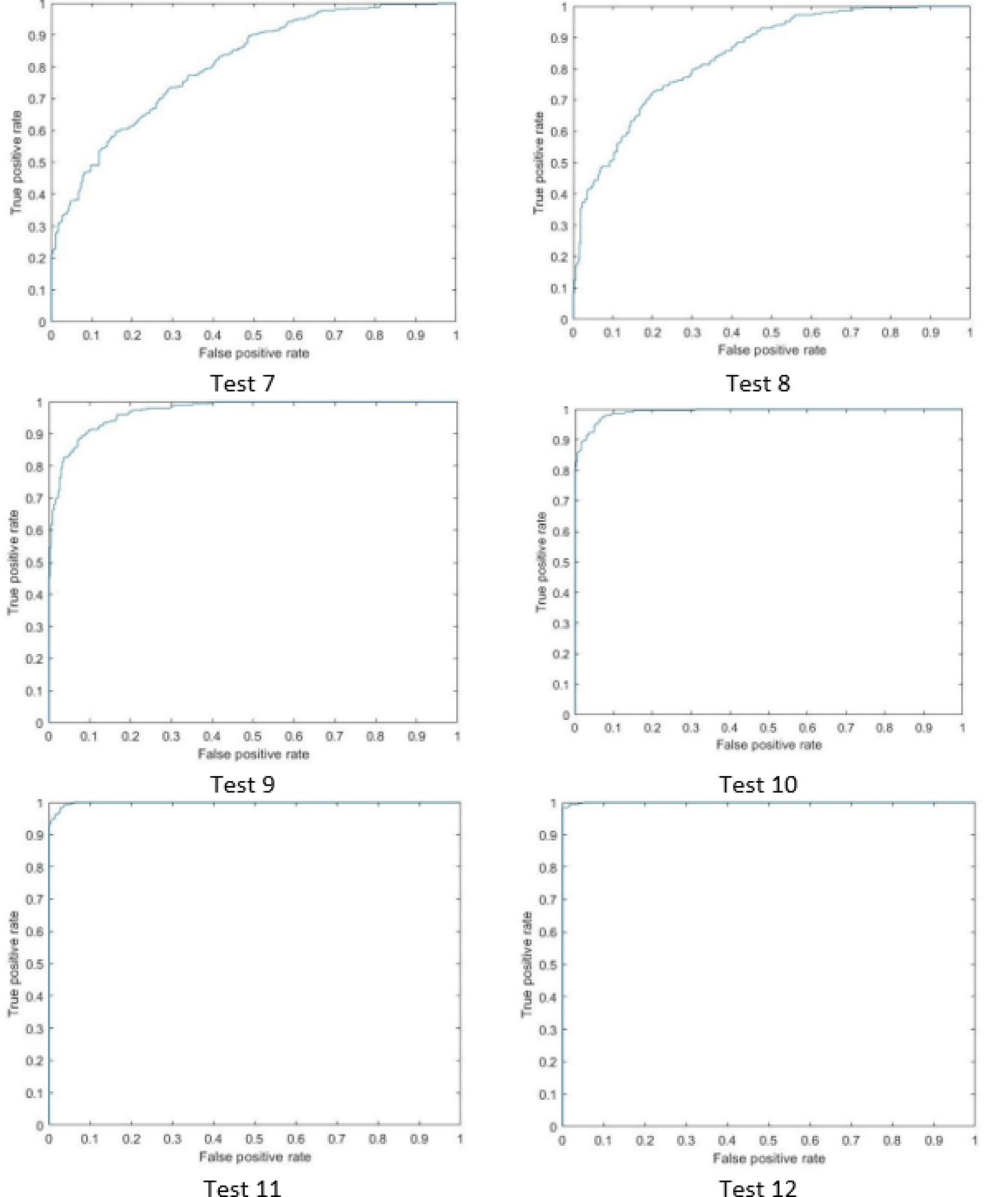
The ROC curve of the second six tests in our proposed method.

The above experiments showed that the range of influence has a high impact on the performance of the model but it takes long time in training and for this reason, squash factor is an alternate choice to reach the good accuracy results and in short time of training. In addition, we noticed that decreasing these two factors, generates more rules but also increases the processing time as it is shown in Fig. 8. An ROC cure for each test is shown in Figs. 9 and 10 and based on the findings of this study, clinicians are highly recommended to apply this technique as an appropriate option for early diagnosis of COVID-19 infection and we hope to be used within the clinical routine in the future.

Despite the good accuracy obtained from using deep learning techniques in automatic detection of COVID-19, we decided to follow a new direction by using the Fuzzy system for classification process which proved its ability to compete the other techniques.Table 2 shows The efficiency of the proposed model compared to the state-of-the-art detection techniques. The performance measures showed a high accuracy that outperform many of the recent techniques used for classifying COVID-19 X-ray images.

## Conclusion

The objective of this research is to use the Adaptive Neuro Fuzzy Inference System (ANFIS) based on subtractive clustering in classification and analyze the effect of its parameters in dealing with large data set of X-ray Images. To achieve this goal, 12 tests have been done until we reached the best performance of our model with 0.9867 accuracy. From these tests, we can conclude that small range of influence will produce large number of rules and more clusters and this results in high accuracy in classification but it takes long time in training which makes it insufficient for real applications, specifically with early diagnosis of diseases like COVID-19. On the other hand, Squash factor also has a valuable effect when its value is small which causes more clusters, and combining it with reasonable value of range of influence factor will lead to high accuracy in very short time.The other two parameters of this classifier which are the accept factor and the reject factor have no noticeable effect on accuracy of classification.

As a future work, it could be possible to try using ANFIS classifier in combination with other feature selection techniques and with optimization techniques to improve its performance specifically in terms of processing time which considered a remarkable limitation when using this classifier with large data set.

**Table 2:** Comparison of the proposed method with the state-of-the-art techniques for binary classification of COVID-19.

References	Technique	Accuracy
Osturk et al.^32^	Darknet	98.08%
Hemdan et al.^34^	COVIDX-Net	60–90%
Narin et al.^35^	ResNet50, InceptionV3 and Inception- ResNetV2)	98%, 97%, 87%
Howart et al.^37^	Shallow CNN	96.92%
Maghdid et al.^38^	Modified AlexNet, simple CNN	94.40%
Asnaoui et al.^39^	CNN, VGG16 VGG19, Inception_V3, Xception, DensNet201, MobileNet_V2, Inception_Resnet_V2,	84.18–96.61%
Saha et al.^41^	EMCNet	98.91%
ANFISNet(ours)	ANFIS + GLCM	98.67%

## Data Availability

We used the updated dataset mentioned by Islam et al.^43^ which is publicly available dataset.

## Code availability

The code of this work will be provided on request.
